# Medical Legislation in Ohio

**Published:** 1894-03

**Authors:** 


### MEDICAL LEGISLATION IN OHIO.

Those of us who live in Georgia may well entertain for our Ohio brethren somewhat of that "fellow-feeling which makes us wondrous kind." We have watched with great interest the fight that has been made by our friends in the Buckeye State for respectable protective medical legislation. In this connection the labors of our bright and energetic contemporary, the Cincinnati *Lancet-Clinic,* are deserving of the highest praise, and we offer herewith our most cordial sympathy and God-speed.

The situation in Ohio is like that in Georgia, if not worse. In both States it is lamentable indeed. Anybody can practice medicine in either place, including the Christian scientist and his congener, the hoodoo charmer. Ohio and Georgia are becoming sadly left behind by other States which are hastening to protect themselves against the hosts of medical ignoramuses and charlatans. Our friends in Ohio are having a hard fight, with the odds against them. From private correspondence we learn that they are debarred the means of presenting their cause directly to the people, from the fact that the newspapers are bought up by the enemy and will not publish anything which may injure the rights of their faithful patrons, the quack doctors. Such a state of affairs is a sad comment upon journalism in Ohio, and for that State at least the "freedom of the press" is what a western senator would call an "iridescent dream."

The Ohio legislature from time to time has been siezed with violent spasms to legislate on matters more or less medical. Not so very long ago it made a fool of itself by appropriating $5,000 to investigate the merits of the Keely cure. It would not surprise us now to see it make a similar exhibition over that other fake, the Amick cure, which is somewhat nearer home. We see it reported that a late Ohio law will forbid circumcision. And why, may we ask, this fierce desire to save a useless foreskin? The present legislature is considering a bill providing that convicted criminals shall be utilized for scientific and physiological experiment and finally put to death by some painless method. This is not so bad. Such is medical legislation in Ohio. The respectable and intelligent medical profession of the State have prayed the legislature to adopt some measure which would protect its citizens against ignorant and dishonest practitioners of medicine, and all their petitions have been ignored. In the meantime Ohio becomes the place of refuge for the army of the great unqualified who are refused a habitation in neighboring States. Shame upon the Pharasaic legislatures of both Ohio and Georgia, who smite their righteous breasts and pay such proper tithe of mint, and anise, and cummin, but neglect the weightier matters of the law!

But time will yet see active and efficient boards in both Ohio and Georgia. We have too much faith in the ultimate triumph of the right to believe otherwise. We again offer our best wishes to our Ohio colleagues, and especially to the *Lancet-Clinic*, reminding them that

> "Thrice is he armed that hath his quarrel just."

